# Adsorption of Cr(VI), Ni(II), Fe(II) and Cd(II) ions by KIAgNPs decorated MWCNTs in a batch and fixed bed process

**DOI:** 10.1038/s41598-020-79857-z

**Published:** 2021-01-08

**Authors:** Titus Chinedu Egbosiuba, Ambali Saka Abdulkareem, Abdulsalami Sanni Kovo, Eyitayo Amos Afolabi, Jimoh Oladejo Tijani, Mercy Temitope Bankole, Shufeng Bo, Wiets Daniel Roos

**Affiliations:** 1 Department of Chemical Engineering, Federal University of Technology, PMB.65, Minna, Niger Nigeria; 2 Department of Chemical Engineering, Chukwuemeka Odumegwu Ojukwu University, PMB 02, Uli, Anambra Nigeria; 3 Department of Chemistry, Federal University of Technology, PMB.65, Minna, Niger Nigeria; 4 Faculty of Light Industry and Chemical Engineering, Dalian Polytechnic University, Dalian, 116034 People’s Republic of China; 5 Department of Physics, University of the Free State, P.O. Box 339, Bloemfontein, 9300 South Africa; 6 Nanotechnology Research Group, Africa Centre of Excellence for Mycotoxin and Food Safety, Federal University of Technology, P.M.B 65, Bosso, Minna, Niger Nigeria

**Keywords:** Chemical engineering, Nanoscale materials

## Abstract

The efficient removal of toxic metals ions from chemical industry wastewater is considered problematic due to the existence of pollutants as mixtures in the aqueous matrix, thus development of advanced and effective treatment method has been identified as a panacea to the lingering problems of heavy metal pollution. In this study, KIAgNPs decorated MWCNTs nano adsorbent was developed using combination of green chemistry protocol and chemical vapor deposition techniques and subsequently characterized using UV–Vis, HRTEM, HRSEM, XRD, FTIR and XPS. The adsorptive efficiency of MWCNTs-KIAgNPs for the removal of Cr(VI), Ni(II), Fe(II), Cd(II) and physico-chemical parameters like pH, TDS, COD, BOD, nitrates, sulphates, chlorides and phosphates from chemical industrial wastewater was examined in both batch and fixed bed systems. The result exhibited successful deposition of KIAgNPs on the surface of MWCNTs as confirmed by the microstructures, morphology, crystalline nature, functional groups and elemental characteristics of the MWCNTs-KIAgNPs. Optimum batch adsorption parameters include; pH (3 for Cr(VI) and 6 for Ni(II), Fe(II) and Cd(II) ions), contact time (60 min), adsorbent dosage (40 mg) and temperature (318 K). The binding capacities were obtained as follows; Cr^6+^ (229.540 mg/g), Ni^2+^ (174.784 mg/g), Fe^2+^ (149.552) and Cd^2+^ (121.026 mg/g), respectively. Langmuir isotherm and pseudo-second order kinetic model best described the experimental data in batch adsorption, while the thermodynamic parameters validated the chemisorption and endothermic nature of the adsorption process. In continuous adsorption, the metal ions were effectively removed at low metal influent concentration, low flow rate and high bed depth, whereby the experimental data were designated by Thomas model. The high physico-chemical parameters in the wastewater were successfully treated in both batch and fixed bed systems to fall within WHO permissible concentrations. The adsorption/desorption study illustrated over 80% metal removal by MWCNTs-KIAgNPs even after 8th adsorption cycle. This study demonstrated excellent performance of MWCNTs-KIAgNPs for chemical industry wastewater treatment.

## Introduction

Wastewater is a major public health problem which are generated from chemical industries such as battery, metal plating, cosmetics, pharmaceuticals, plastic and textiles^1–3^. The wastewater contains various pollutants, including potentially toxic metals which are detrimental to humans, animals and aquatic organisms. Heavy metals such as Cd(II), Cr(VI) Fe(II) and Ni(II) are increasingly recognized as a serious global threat to public health due to their non-biodegradability, mobility, toxicity and bioaccumulation potentials in the liver, kidney and other human bodies^4,5^. For instance, chronic exposure to cadmium have adverse impact on human bone, kidney, increases blood pressure and causes cancer^6^. An increase in exposure to chromium results to cancer, asthma, diarrhea. Liver defect, kidney problems and brain defect leading to physiological impairment^7,8^. The presence of iron above tolerable limit in wastewater have been reported to be carcinogenic and causes intestinal damage and irritation of respiratory tract^9^. While long term exposure to nickel is also carcinogenic and a major cause of brain hemorrhage, cardiac arrest, asthma, dermatitis, liver and heart damage^10^. Therefore, the primary concern of wastewater treatment experts is to mitigate the presence of these heavy metals in aqueous matric even in trace amounts. Recent developments on the non-degradability and toxicity of these heavy metals have heightened the interests in the removal of these heavy metals from industrial wastewater^4^.

Recently, researchers have examined the removal of heavy metals using numerous techniques, including ion exchange, chemical precipitation, reverse osmosis, membrane processes, microbial biotechnology, coagulation, flocculation, filtration and adsorption technology^11^. However, these techniques possess different limitations such as low efficiency, high cost, generation of toxic byproducts, delay in operation, inefficiency in targeting specific pollutants and complexity of treatment methods^12^. Of these techniques, adsorption technology has been identified as one of the most efficient and commonly used treatment method due to its ease of operation control, regeneration potentials, cost efficient, inertness to materials, lack of sludge formation and variety of adsorbents^13–15^. In recent years, nanomaterials have attracted the attention of researchers due to their unique physicochemical properties attributable to their small size, shape, dimensions, large surface area, crystallinity and composition^16^. These foremost properties enhanced the suitability of nanomaterials application in water treatment, catalysis, medicine and biotechnology^12^. At the moment, nanomaterials-based adsorbents such as zinc oxide, tin oxide, graphene oxide, carbon nanotubes, silica, aluminum oxide, titanium oxide, zeolites, iron oxide, spinel ferrites, chitosan, carbon nanofibers and cerium oxide have been employed by different researchers for industrial wastewater treatment^17–25^. Among these nanomaterials, carbon nanotubes (CNTs) have been extensively exploited due to their large surface area, ease of modification, extraordinary surface chemistry, structural control, low density, porosity, higher thermal stability, high chemical stability, regeneration ease and reusability, compared to other adsorbents^26^. Previous studies have reported increase in the adsorption capacity of CNTs in the uptake of heavy metals from wastewater after oxidation with either acid or base^23^. Similarly, the successful interaction of the oxidized CNTs with the heavy metals and even organic compounds have been attributed to their surface functional groups and hydrophobic surfaces through electrostatic and hydrogen bonding^22^.

In view of the interaction of the surface chemistry of CNTs, some studies have investigated the incorporation of metallic nanoparticles to the surface of CNTs due to their distinctive properties such as antimicrobial activities, catalysis, electricity conduction and high stability for chemicals^27^. Metal nanoparticles such as copper (Cu), iron (Fe), cobalt (Co), nickel (Ni), palladium (Pd), gold (Au), platinum (Pt) and silver (Ag) are frequently used^28,29^. Among the metallic nanoparticles (NPs), AgNPs are the most widely utilized in the removal of heavy metals, organic matters, dyes and antibiotics from wastewater. AgNPs have been synthesized through different chemical and physical methods which are not eco-friendly due to hazardous chemical utilization, expensive and operated at high temperature. Recent evidence suggests that efforts to mitigate the increased environmental challenges of the chemical techniques enhanced the development of green synthesis of AgNPs using plant materials, fungi, algae and bacteria^30^. Of these, plant extracts have been dominantly utilized because of its low cost, less toxicity, improved structural control and lack of pre-treatment methods^28^. The plant extracts contain various phytochemicals such as flavonoids and polyphenolic substances which facilitates the reduction of silver salt to zerovalent silver NPs and also protects it from agglomeration. Similarly, plant extracts and plant derivatives such as *Koelreuteria apiculate*^30^, starch solution^12^, *Nauclea latifolia*^31^ and *Diospyros lotus*^32^ have been used to synthesize AgNPs via green chemistry. To the best of our knowledge, no study has reported the green synthesis of AgNPs using *Khaya ivorensis* (African mahogany) leaf extract.

In the current study, *Khaya ivorensis* (KI) plant extract was utilized to synthesize KIAgNPs for the decoration of MWCNTs. The developed MWCNTs-KIAgNPs were characterized and the efficiency of MWCNTs-KIAgNPs for the adsorption of Cd(II), Cr(VI), Fe(II) and Ni(II) from industrial wastewater was investigated in a batch and dynamic flow processes. Overall, continuous column adsorption is the most favorable system for potential practical applications of developed adsorbent in wastewater treatment. In spite of the fixed bed column adsorption significance, there is paucity of information on the removal of Cd(II), Cr(VI), Fe(II) and Ni(II) using MWCNTs-KIAgNPs. The influence of pH, adsorbent dosage, temperature and contact time on the metal adsorption were explored for the batch mode, while the influent concentration, flow rate and bed depth were examined for the continuous flow adsorption.

## Materials and methods

### Materials

Sodium hydroxide (NaOH), Silver nitrate (AgNO_3_, 99.9%), dimethylformamide (DMF, 99%) and hydrochloric acid (HCl, 99%) of analytical grade were purchased from Sigma Aldrich and used as received without further purification. Deionized water obtained by Milli-Q system was used throughout the research. Healthy leaves of *Khaya ivorensis* (KI) were collected from Bosso campus of Federal University of Technology Minna, Nigeria. The identification and authentication of the plant was properly done prior to deposition of the sample in the university herbarium with the sample number FUT/STEP-B/T005. The wastewater was collected from the clusters of chemical industries at industrial layout Nnewi, Anambra Nigeria. The MWCNTs used in this study were synthesized as reported in our previous study^33^.

### Preparation of KI leaf extract

To prepare the plant extract, KI leaves were washed with distilled water followed by deionized water. 100 g of the KI leaves were cut into small pieces and boiled with 1000 mL of deionised water at 60 °C for 30 min to obtain aqueous extract. Thereafter, the KI extract was cooled, filtered by Whatman No. 1 filter paper and stored at 40 °C for further application in biosynthesis of KIAgNPs.

### Phytochemical analysis

Herein, main secondary metabolites in plant extract that enhances the formation of nanoparticles were identified by determining the presence of phytochemicals like tannins, phenols, alkaloids, saponins, flavonoids, steroids and terpenoids. Initially, the presence of tannins was confirmed by the appearance of greenish black or dark blue as 2 mL of 5% ferric chloride was added to 1 mL of KI extract^34^. Phenols were identified by the addition of 2 mL of 5% ferric chloride into 0.2 g of the plant extract and the formation of a greenish-brown or black precipitate color indicate presence for phenols^32^. Also, alkaloids presence in the KI extract was revealed if a white or green precipitate appears on addition of few drops of Mayers reagent into a mixture containing 2 mL each of the KI extract and dilute hydrochloric acid^32^. In order to determine the presence of saponins, about 0.2 g of KI leaves were added to 5 mL of distilled water, shaken for some time and heated to boil. Then saponins were confirmed to be present with a stable foam (froth) appearance^35^. The flavonoids were examined by treating the KI extract (1 mL) with 10% NaOH solution (1 mL), while the appearance of intense yellow precipitate confirmed the presence of flavonoids^34^. About 0.5 g of dried KI extract was dissolved in 2 mL of acetic anhydride solution before adding concentrated sulfuric acid (2 mL), then the occurrence of steroids was confirmed with color change from violet to green or blue^32^. The determination of terpenoids was achieved by mixing dried extract (0.1 g) with chloroform (0.5 mL) prior to careful introduction of concentrated sulfuric acid (1 mL) to form a layer. The presence of a reddish brown color at the interface confirmed the formation of terpenoids^35^.

Subsequently, the quantification of the total tannins, phenols, alkaloids and flavonoids in the aqueous KI leaf extract were determined through validated methods and standard procedures^32,34–36^.

### Optimization of green synthesis of KIAgNPs

In this study, the influence of various parameters such as temperature, pH and volume of KI extract to silver nitrate solution were investigated using factorial design of experiment shown in Table 2a for high and low values of the parameters. Notably, green synthesis of KIAgNPs was carried out in a dark room using an ultrasonic bath (SB25-12DT, Ultrasonic Scientz). As shown in Table 2b, 80 mL of KI extract and 20 mL of silver nitrate (0.05 M) solution was measured into a 250 cm^3^ conical flask maintained at the pH of 8 (using HCl and NaOH) and placed inside the ultrasonic bath set at 70 °C. As the temperature was achieved, the KI extract was added to the AgNO_3_ solution with the heating maintained for 15 min. Thereafter, the reduction of silver ions (Ag^+^) to silver (Ag^0^) nanoparticles was observed through color change from yellow to deep brown^37^. Also, the wavelength of the sample was determined using UV- visible spectroscopy and the same procedure was repeated for runs 2 to 8 as indicated in Table 2b.

### Decoration of MWCNTs with KIAgNPs

In this study, MWCNTs-KIAgNPs composite were prepared in two phases to incorporate carboxyl and hydroxyl functional groups and to enhance the adsorption properties of MWCNTs with KIAgNPs.

Herein, MWCNTs-KIAgNPs composite were prepared in two phases to incorporate carboxyl and hydroxyl functional groups and to enhance the adsorption properties of MWCNTs with KIAgNPs. In the first phase of the nano adsorbent fabrication, 25 g of MWCNTs was mixed with 200 and 600 mL of nitric acid and sulfuric acid in order to incorporate negatively charged functional groups to the MWCNTs surface^38^. Thereafter, the mixture was placed in ultrasonic bath set at 40 °C for 3 h to add carboxyl and hydroxyl groups onto the surface of MWCNTs. Subsequently, the mixture was washed with deionized water to a neutral pH and filtered using Whatman grade 1 filter paper. In the end, the functionalized MWCNTs was oven dried at 110 °C for 12 h.

Furthermore, the second phase is the enhancement of the properties of functionalized MWCNTs using KIAgNPs. Herein, 20 g of the MWCNTs was dispersed in a solution containing 50 mL of DMF and 100 mL KIAgNPs and sonicated for 3 h to enhance the dispersion of the carbon nanotubes with the homogenous blend of the silver nanoparticles. Thereafter, the blend of MWCNTs-KIAgNPs was washed with distilled water until a neutral pH was achieved for the washed water. Subsequently, the obtained MWCNTs-KIAgNPs was oven dried at 110 °C for 12 h. Following this, the MWCNTs-KIAgNPs hybrid was stored in an airtight container for further characterization and used as nano adsorbent for the uptake of Cd(II), Cr(VI), Fe(II) and Ni(II) from chemical industrial wastewater in a batch and fixed bed process.

### Characterization of KIAgNPs and MWCNTs-KIAgNPs

The formation and optimization of KIAgNPs were studied using a UV–visible spectroscopy (Cary100, Agilent Technologies, USA). The KIAgNPs and MWCNTs-KIAgNPs were characterized for their microstructures using high resolution transmission electron microscope (HRTEM, Zeiss Auriga). The morphological analysis of MWCNTs-KIAgNPs was examined by high resolution scanning electron microscopy (HRSEM, Zeiss Auriga). The MWCNTs-KIAgNPs surface area was examined using Brunauer–Emmett–Teller (NOVA4200e, Quantachrome UK), while their phase structures were analyzed using X-ray diffraction (XRD, 6000, Shimadzu Scientific). Additionally, the measurement of the zeta potential was carried out using a zetaView PMX100 micro-electrophoretic instrument (Particle Metrix GmbH, Meerbusch, Germany)). The point of zero charge (PZC) was determined by titration method, where MWCNTs-KIAgNPs of 0.04 g were added to deionized water. The pH meter (Eutech Instruments) was utilized for solutions charge densities determination, while the 0.05 M of HNO_3_ and NaOH was used to adjust the surface charge of MWCNTs-KIAgNPs in the pH range of 2–8 until a zero charge density was obtained.

### Adsorption performance evaluation

#### Metal analysis and physicochemical characterization

Herein, the presence of metal ions such as Cr(VI), Ni(II), Fe(II) and Cd(II) in the chemical industry wastewater were evaluated using atomic absorption spectrometer (AAS, PG 900 Instruments, UK) method after proper dilution. Standard techniques were particularly used for physicochemical analysis of the wastewater before and after treatment in a batch and dynamic column mode. The pH, total dissolved solids (TDS), chemical oxygen demand (COD) and biochemical oxygen demand (BOD) were determined by pH meter, gravimetric procedure, 5 days technique and filtrate drying technique^39,40^. Further, the nitrates, sulphates, chlorides and phosphates were determined by brucine reagent, gum acacia, argentometric titration and ammonium molybdate reagent^40,41^.

#### Batch adsorption studies

Batch adsorption parameters such as pH, contact time, amount of MWCNTs-KIAgNPs and temperature were duly optimized to enable improved removal of Cd(II), Cr(VI), Fe(II) and Ni(II) from chemical industrial wastewater. In this study, the influence of adsorption parameters on metal ions removal were varied as follows; pH (2 to 8, altered using 0.1 mol/L NaOH or HNO_3_), contact time (10 to 120 min), amount of MWCNTs-KIAgNPs (10 to 60 mg) and temperature (303 to 318 K). Initially, 10 mg of MWCNTs-KIAgNPs was added to 100 mL of wastewater solution containing 44, 85, 56 and 66 mg/L of Cd(II), Cr(VI), Fe(II) and Ni(II) in a sealed conical flask (200 mL) and stirred continuously (150 rpm) in a thermostatic bath for 10 min at a pH and temperature of 5 and 40 °C, respectively. Thereafter, the sample was filtered using Whatman grade 1 filter paper and the filtrate analyzed for the remaining adsorbate amount by atomic adsorption spectrophotometer (AAS, PG 990, PG Instruments, UK). Similarly, the optimized process parameters were achieved using the same procedure to investigate the adsorption conditions on the removal rate of Cd(II), Cr(VI), Fe(II) and Ni(II) ions by MWCNTs-KIAgNPs.

The rate of removal (R) and the adsorption capacity (q_t_ and q_e_ (mg/g)) were evaluated using the following mathematical expressions^42,43^.

1 $$\mathrm{R}= \frac{{\mathrm{C}}_{0}-{\mathrm{C}}_{\mathrm{e},\mathrm{ t}}}{{\mathrm{C}}_{0}}\mathrm{ x }100\mathrm{\%}$$

2 $${\mathrm{q}}_{\mathrm{t }}= \frac{\left({\mathrm{C}}_{0}-{\mathrm{C}}_{\mathrm{t}}\right)\mathrm{V}}{\mathrm{m}}$$

3 $${\mathrm{q}}_{\mathrm{e }}= \frac{\left({\mathrm{C}}_{0}-{\mathrm{C}}_{\mathrm{e}}\right)\mathrm{V}}{\mathrm{m}}$$

in which q_t_ and q_e_ refers to the adsorption capacity of metal ions at t (min) and equilibrium time, respectively. C_0_ (mg/L) is the initial concentration, while C_e_ (mg/L) and C_t_ (mg/L) denotes the equilibrium and t time concentration of metal ions, respectively. Also, m (mg) and V (L) are the amount of adsorbent and volume of adsorbate solution.

Further evaluation of the static adsorption behavior of Cd(II), Cr(VI), Fe(II) and Ni(II) ions by MWCNTs-KIAgNPs was conducted using Langmuir^44^ and Freundlich^10^ adsorption isotherm models described in Eqs. (4) and (5), respectively.

4 $$\frac{{C}_{e}}{{\mathrm{q}}_{\mathrm{e}}}=\frac{1}{{\mathrm{K}}_{\mathrm{L}}{\mathrm{q}}_{\mathrm{m}}}+ \frac{{\mathrm{C}}_{\mathrm{e}}}{{\mathrm{q}}_{\mathrm{m}}}$$

5 $${\mathrm{Inq}}_{\mathrm{e}}=\mathrm{ In }{\mathrm{K}}_{\mathrm{F}}+ \frac{1}{{\mathrm{n}}_{\mathrm{F}}}{\mathrm{InC}}_{\mathrm{e}}$$

in which the maximum adsorption capacity and Langmuir coefficient are denoted by q_m_ (mg/g) and K_L_ (L/mg). Also, K_F_ is the Freundlich equilibrium constant, representing the degree of the adsorption capacity, while n_F_ is the heterogeneity factor that measures the strength of adsorption.

In addition, pseudo first-order and pseudo second-order models were utilized to fit the kinetic data using the relationships expressed in Eqs. (6) and (7)^42^, respectively.

6 $$\mathrm{In }({\mathrm{q}}_{\mathrm{e}}- {\mathrm{q}}_{\mathrm{t}})=\mathrm{In }{\mathrm{q}}_{\mathrm{e}}- {\mathrm{k}}_{1}\mathrm{t}$$

7 $$\frac{\mathrm{t}}{{\mathrm{q}}_{\mathrm{t}}}= \frac{1}{{\mathrm{k}}_{2}{\mathrm{q}}_{\mathrm{e}}^{2}}+ \frac{\mathrm{t}}{{\mathrm{q}}_{\mathrm{e}}}$$

in which k_1_ (min^−1^) and k_2_ (g/mg min) represent the rate constants for the pseudo-first-order and pseudo-second-order kinetic model.

Also, thermodynamic parameters for instance; Gibbs free energy (ΔG°), enthalpy (ΔH°) and entropy (ΔS°) were evaluated to establish the nature of Cd(II), Cr(VI), Fe(II) and Ni(II) ions capture by MWCNTs-KIAgNPs using Eqs. (8) and (9)^45^.

8 $$\mathrm{\Delta G}^\circ =\mathrm{ \Delta H}^\circ -\mathrm{T\Delta S}^\circ$$

9 $$\mathrm{In }\frac{{\mathrm{q}}_{\mathrm{e}}}{{\mathrm{C}}_{\mathrm{e}}}= - \frac{\mathrm{\Delta H}^\circ }{\mathrm{RT}}+\frac{\mathrm{\Delta S}^\circ }{\mathrm{R}}$$

in which ΔHº (J mol^−1^) and ΔSº (J K^−1^ mol^−1^) refers to the changes in enthalpy and entropy, while R and T are the gas constant (8.314 JK^−1^ mol^−1^) and temperature (K), respectively. The linear plot of $\mathrm{In }\frac{{\mathrm{q}}_{\mathrm{e}}}{{\mathrm{C}}_{\mathrm{e}}}$ against $\frac{1}{\mathrm{T}}$ assisted the determination of ΔHº (kJ mol^−1^) and ΔSº (kJ K^−1^ mol^−1^) as the slope and intercept, respectively.

#### Column adsorption experiments

The fixed bed adsorption behavior was examined in a glass column of diameter (3 cm), height (30 cm) and volume (250 mL) to investigate the treatment capacity of MWCNTs-KIAgNPs in a large scale. Particularly, the adsorption column was filled with 0.8 g of MWCNTs-KIAgNPs (average size (8.73 nm), bed height (2, 4 and 6 cm)), while both ends of the glass column were covered with gauze and cotton wool to enhance wastewater homogenous dispersion. The wastewater containing metal ions at the following initial concentrations (Cd(II) (11, 22 and 44 mg/L), Fe(II) (14, 28 and 56 mg/L), Cr(VI) (21.25, 42.5 and 85 mg/L) and Ni(II) (16.5, 33 and 66 mg/L)) were pumped into the dynamic glass column using a peristaltic pump from bottom to top at various flow rates (5, 7.5 and 10 mL/min). Subsequently, the effluent from the column was collected with vials at various time intervals and the metal ions concentration determined by AAS technique. Notably, the plot of breakthrough curves was based on normalized concentration at a specified time to initial concentration (C_t_/C_o_) against time profile^46^. Accordingly, the breakthrough time (*t*_b_) and exhaustion time (*t*_e_) were evaluated at the C_t_/C_o_ value equivalence of 5 and 95%, respectively^42,47^. Above all, the capacity of wastewater treatment (V_b_, mL; V_total_, mL and m_total_ mg) was calculated using Eqs. (10), (11) and (12)^42^.

10 $${\mathrm{V}}_{\mathrm{b}}={\mathrm{Qt}}_{\mathrm{b}}$$

11 $${\mathrm{V}}_{\mathrm{total}}={\mathrm{Qt}}_{\mathrm{total}}$$

12 $${\mathrm{m}}_{\mathrm{total}}= \frac{{\mathrm{C}}_{\mathrm{o}}{\mathrm{Qt}}_{\mathrm{total}}}{1000}$$

The total amount of metal ions adsorbed (q_total_, mg) was determined by the area under the (C_o_-C_t_) ~ t curve indicated in Eq. (13), while the maximum adsorption capacity of MWCNTs-KIAgNPs (q_e_, mg/g) was evaluated with the formula expressed in Eq. (14)^47^.

13 $${\mathrm{q}}_{\mathrm{total}}= \frac{\mathrm{Q}}{1000}\underset{\mathrm{t}=0}{\overset{\mathrm{t}= {\mathrm{t}}_{\mathrm{total}}}{\int }}({\mathrm{C}}_{\mathrm{o}}- {\mathrm{C}}_{\mathrm{t}})\mathrm{dt}$$

14 $${\mathrm{q}}_{\mathrm{e}}= \frac{{\mathrm{q}}_{\mathrm{total}}}{\mathrm{m}}$$

Meanwhile, the total metal ions adsorption (R_total_, %) and the contact time of empty column (CTEC, min) were determined using Eqs. (15) and (16), respectively^42^.

15 $${\mathrm{R}}_{\mathrm{total}} (\mathrm{\%})= \frac{{\mathrm{q}}_{\mathrm{total}}}{{\mathrm{m}}_{\mathrm{total}}}\mathrm{ x }100$$

16 $$\mathrm{CTEC}= \frac{\mathrm{bed volume}}{\mathrm{Q}}$$

in which Q is the flow rate (mL/min), while t_b_ (min) and ${\mathrm{t}}_{\mathrm{total}}$ (min) represents the time at breakthrough and saturation point, respectively. Also, ${\mathrm{V}}_{\mathrm{b}}$ and ${\mathrm{V}}_{\mathrm{total}}$ are the volume of wastewater treated at breakthrough and the total wastewater treated.

To further predict the dynamic behavior of Cd(II), Cr(VI), Fe(II) and Ni(II) ions adsorption by MWCNTs-KIAgNPs, the experimental data were fitted by Adams-Bohart (Eq. (17)) and Thomas (Eq. (18)) models to typically describe the breakthrough curves^48,49^.

17 $$\frac{{C}_{t}}{{C}_{o}}= {e}^{{K}_{AB}{C}_{0}t - {K}_{AB}{{N}_{0}}_{F}^{Z}}$$

18 $$\frac{{C}_{t}}{{C}_{o}}= \frac{1}{1+ {e}^{\frac{{K}_{T}{q}_{0}m}{Q} - {K}_{T}{C}_{0}t}}$$

where K_AB_ (L/(min mg) and K_T_ (L/(min mg)) represents Adams-Bohart and Thomas kinetic constant, while N_0_ (mg/L), F (cm/min) and Z (cm) refers to the concentration at saturation, linear velocity and depth of the column, respectively.

### Batch/column regeneration tests

In order to evaluate the regeneration capacity of MWCNTs-KIAgNPs after the metal ions adsorption in a batch and continuous approach, the used nano adsorbent (20 mg) was treated using 0.5 M H_2_SO_4_ (50 mL) in a 250 mL conical flask. The solution was continuously stirred (150 rpm) at 40 °C for 2 h. Thereafter, Whatman grade 1 filter paper was utilized to filter the mixture, followed by washing with deionized water to achieve pH 7. The regenerated nano adsorbent was dried to constant weight prior to subsequent usage. Herein, the nano adsorbent performance studies were repeated 5 cycles and the effectiveness (η) determined using Eq. (19)^50^.

19 $$\upeta =\frac{\mathrm{Co}-\mathrm{ Ce}}{\mathrm{Co}} \times 100\mathrm{ \%}$$

in which C_o_ (mg/L) and C_e_ (mg/L) are the initial and equilibrium concentration of the metal ions, respectively.

## Results and discussion

### Qualitative and quantitative phytochemical determination

For further evaluation of the qualitative and quantitative presence of phytomolecules in KI leaf extract, phytochemical screening was carried out and the result presented in Table 1.

**Table 1:** Phytochemical analysis of *Khaya ivorensis* (KI) leaf extract.

Phytochemical	Qualitative	Quantitative (mg/g)
Alkaloids	+	31.66 ± 0.03
Phenols	+	102.23 ± 0.07
Tannins	+	5.14 ± 0.01
Flavonoids	+	20.94 ± 0.05
Saponins	+	25.15 ± 0.02
Terpenoids	+	3.84 ± 0.04
Steroids	−	ND
Glucosides	+	ND

Key: +  = present; − = absent.

*ND* not determined.

As shown in Table 1, the results revealed the presence of alkaloids, phenols, tannins, flavonoids, saponins, glucosides and terpenoids, while steroids and anthraquinones were absent. Moreso, the quantitative presence of the following phytomolecules; alkaloids (31.66 mg/g), phenols (102.23 mg/g), tannins (5.14 mg/g), flavonoids (20.94 mg/g), saponins (25.15 mg/g) and terpenoids (3.84 mg/g) were also identified. The result of the phytochemical screening revealed high concentration of biomolecules responsible for effective Ag^+^ bio-reduction to Ag^0^ and enhance the stabilization of KIAgNPs^31,51^.

### Optimization of green synthesis of KIAgNPs

**Figure 1:**
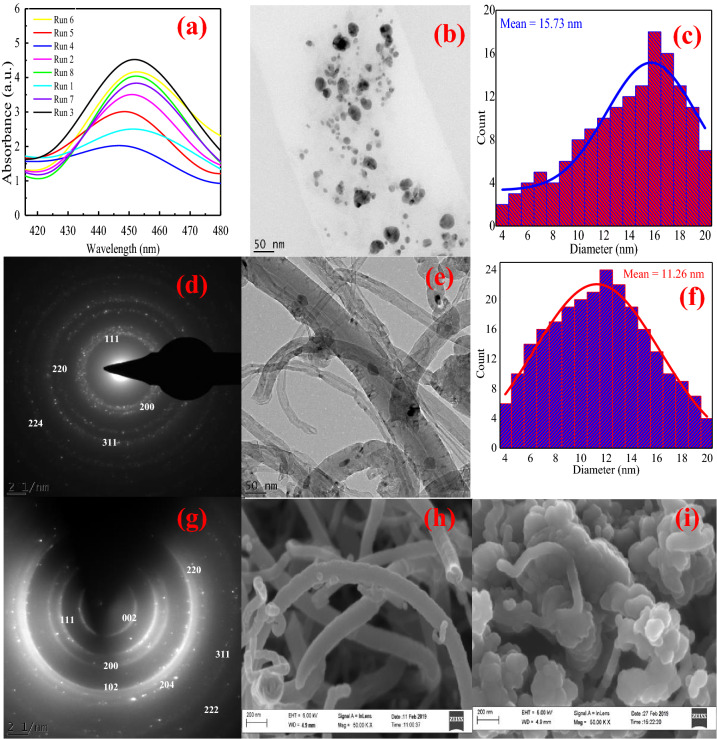
**(a)** UV–Vis spectra of 2^3^ factorial design optimization of KIAgNPs; HRTEM image of **(b)** KIAgNPs; **(c)** diameter distribution of KIAgNPs; **(d)** SAED pattern of KIAgNPs; HRTEM image of **(e)** MWCNTs-KIAgNPs; **(f)** Diameter distribution of MWCNTs-KIAgNPs; **(g)** SAED pattern of MWCNTs-KIAgNPs; HRSEM of MWCNTs-KIAgNPs **(h)** before adsorption and **(i)** after adsorption.

A 2^3^ factorial design of experiment was used to investigate the effect of synthesis parameters such as temperature, pH and volume of AgNO_3_ to KI extract, while the result is shown in Table 2c. According to Table 2c, optimum formation of KIAgNPs at the wavelength of 450 nm was obtained at the temperature (70 °C), pH (10) and volume of AgNO_3_ to KI extract (2:8), respectively. Although the formation of KIAgNPs was observed visually through color change from yellow to brown, but UV–Vis spectroscopy excellently confirmed bio-reduction reaction of KIAgNPs due to the surface plasmon resonance (SPR) band excitation in the UV–Vis region. The UV–Visible spectra of the biosynthesis of KIAgNPs using 2^3^ factorial design is displayed in Fig. 1a.

**Table 2:** (a) 2^3^ factorial design matrix; (b) detailed experimental run for green synthesis of KIAgNPs and (c) wavelength response of green synthesized KIAgNPs.

(a)				
Coded values	Temperature (°C)	pH	Volume of KI extract to Ag^+^ solution (mL)	
− Level	40	8	2:8	
+ Level	70	10	8:2	

(b) Run				
	Temperature (°C)	pH	Volume of KI extract to Ag^+^ solution (mL)	
1	40	8	2:8	
2	70	8	2:8	
3	40	10	2:8	
4	70	10	2:8	
5	40	8	8:2	
6	70	8	8:2	
7	40	10	8:2	
8	70	10	8:2	

(c)				
Run	Temperature (°C)	pH	Volume of KI extract to Ag^+^ solution (mL)	Wavelength (nm)
1	70	8	8:2	448
2	70	8	2:8	449
3	70	10	2:8	450
4	40	8	8:2	447
5	40	10	8:2	447
6	40	10	2:8	449
7	40	8	2:8	448
8	70	10	8:2	447

The result presented in Fig. 1a revealed that the absorbance peak occurred between 447 to 450 nm at the various process conditions, indicating the formation of KIAgNPs. In all, the optimization of biosynthesis parameters showed that temperature increase lead to increment in SPR band and decrease in KIAgNPs mean diameter^37^. In addition, pH increase to 10 resulted to high absorption peak, indicating that -OH groups influences the bioreduction of Ag^+^ to Ag^0^ in a basic medium^51^. The capping, reduction and stabilization effects of KI leaves extract further demonstrated the use of Ag^+^ solution in excess to ensure well dispersed and agglomerated free KIAgNPs.

Typically, temperature (70  °C), pH (10) and volume of KI leaf extract to Ag^+^ solution (2:8) showed the most intense peak at 450 nm. Statistical significance of KIAgNPs synthesis parameters was analyzed using of variance (ANOVA). As shown in Table S1, pH exerted the highest influence on biosynthesis of KIAgNPs with F-value of 16.77, followed by volume of KI extract to AgNO_3_ solution and temperature with F-values of 12.42 and 4.36, respectively. The results on the biosynthesis of KIAgNPs obtained in this study corroborated the findings reported previously by Tripathi et al.^52^ and Hamedi et al.^32^ using *Withania coagulans* and *Diospyros lotus* leaf extract, respectively. Over the literature, it was previously reported that the peak of absorbance for AgNPs occur within the range of 400 to 450 nm^37,53^. Hence, the results of this study confirm the bio synthesis of KIAgNPs of various particle sizes and shapes which could be ascribed to the differences in the observed SPR peaks^53^.

### Characterization of KIAgNPs and MWCNTs-KIAgNPs

HRTEM technique examined the microstructure, morphology, size and crystalline nature of KIAgNPs and MWCNTs-KIAgNPs and the results are shown in Fig. 1b,e. From Fig. 1b, the HRTEM image of KIAgNPs exhibited a typical spherical and an ellipsoidal morphology demonstrating loosely bound particles. In addition, Fig. 1e revealed the deposited KIAgNPs on the surface of tubular network of MWCNTs. The HRTEM analysis of KIAgNPs and MWCNTs-KIAgNPs were measured in 50 nm range and the particle size distribution by histogram curves presented in Fig. 1c,f. An average particle size of 15.73 and 11.26 nm was obtained for KIAgNPs and MWCNTs-KIAgNPs which showed consistency with the evaluated particle size of XRD pattern using Debyne Scherrer equation.

In Fig. 1d,g, the selected area of electron diffraction pattern (SAED) revealed bright ring patterns presence indicating the crystalline nature of the KIAgNPs and MWCNTs-KIAgNPs due to the different planes. Correspondingly, these patterns revealed fringes with bright round rings associated with (002), (111). (102), (200), (220), (204), (311) and (222) of Bragg’s planes which also validate the crystallinity of the KIAgNPs and MWCNTs-KIAgNPs^54^. The SAED pattern results showed good agreement with the XRD analysis, thereby suggesting the crystalline nature of the KIAgNPs and MWCNTs-KIAgNPs. The elemental chemical distribution of MWCNTs-KIAgNPs determined by energy dispersive X-ray spectrometer (EDS) is presented in Fig. S1. As observed from the result, strong signal of graphitic carbon and elemental silver nanoparticles were observed, which confirmed the successful incorporation of KIAgNPs onto the surface of MWCNTs. The presence of other components could be attributed to the KI leaf extract and analysis assays as reported over the literature^37,54^.

The surface morphology of the MWCNTs-KIAgNPs were observed before and after adsorption using HRSEM as shown in Fig. 1h,i. Accordingly, the HRSEM image in Fig. 1h revealed the successful incorporation of a well distributed asymmetrical and spherical KIAgNPs on the surface of MWCNTs forming a tubular, intertwined network of MWCNTs-KIAgNPs structure. The cylindrical nanostructure of natural alignment transformed into ropes bound together by Van der Waals forces. Moreover, aggregated KIAgNPs were formed on the surface of the MWCNTs evident from the observed bright contrast on the surface of MWCNTs. Accordingly, the EDS elemental compositions presented in Table S2 revealed successful decoration of MWCNTs using KIAgNPs, which corresponds to the result of HRTEM analysis (Fig. S1). Typically in Fig. 1i, the morphology of MWCNTs-KIAgNPs slightly changed after adsorption process. An apparent aggregation of the chain-like morphology was observed with a slight reduction in the entangled tubular network of MWCNTs. The observed changes could be ascribed to the attachment of the adsorbed metal ions onto the surface of the MWCNTs-KIAgNPs as depicted by the EDS (Table S3). Similar observation was reported by Moazzen et al.^38^ in the adsorption of phthalic acid esters (PAEs) from carbonated soft drinks using MWCNT-Fe_3_O_4_/Ag.

**Figure 2:**
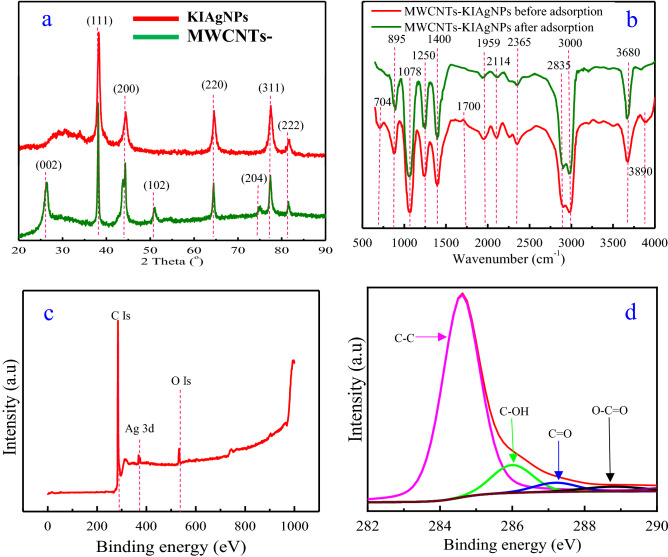
XRD of **(a)** KIAgNPs and MWCNTs-KIAgNPs; **(b)** FTIR of MWCNTs-KIAgNPs before and after adsorption; **(c)** XPS full survey scan spectra of MWCNTs-KIAgNPs; **(d)** high resolution C 1s spectra of MWCNTs-KIAgNPs.

Furthermore, the XRD patterns of the synthesized KIAgNPs and MWCNTs-KIAgNPs are shown in Fig. 2a. The occurrence of characteristic crystalline peaks of AgNPs were observed at 2θ values of 38.32º, 44.50º, 64.59º, 77.55º and 81.81º. These observed diffraction peaks correspond to the following lattice planes (111). (200), (220), (311) and (222) of a typical face-centered cubic (FCC) structure of metallic silver crystal. The crystalline peaks of KIAgNPs in this study revealed significant agreement with other XRD patterns in the literature^37,55^. It can also be seen from the XRD pattern of MWCNTs-KIAgNPs in Fig. 2a that diffraction peaks were observed at the 2 theta values; 26.55º, 38.32º, 44.50º, 50.97º, 64.59º, 74.65º, 77.55º and 81.81º. According to our previous study, the diffraction peaks for MWCNTs occurred at the 2 theta values of 26.55º, 50.97º and 74.65º typical for graphite carbon which were ascribed to the crystal planes (002), (102) and (204)^33^. It is apparent from the XRD pattern of MWCNTs-KIAgNPs that the diffraction peaks at 38.32º, 44.50º, 64.59º, 77.55º and 81.81º indicate that KIAgNPs were strongly deposited on the surface of the MWCNTs^27,38^. The crystal sizes of the KIAgNPs and MWCNTs-KIAgNPs was determined using Debye–Scherrer equation stated in Eq. (20)^54^.

20 $$\mathrm{D}= \frac{\mathrm{K\lambda }}{\mathrm{\beta cos\theta }}$$

in which D (nm) represent the crystal size, while K denotes the shape factor (0.94). The parameters, λ (nm) and β (radians) are the X-ray wavelength (0.154 nm) and full width at half maximum (FWHM). Also, θ (degree) denotes the Bragg's angle. On the average, the crystallite sizes of the KIAgNPs and MWCNTs-KIAgNPs were 15.74 and 11.30 nm respectively and showed similarity to the result of HRTEM analysis in Fig. 1b.

Additionally, the evaluation of the surface properties of MWCNTs-KIAgNPs using BET technique demonstrated the values of 1236 ± 5.40 m^2^/g, 0.74 ± 0.02 cm^3^/g and 28 ± 0.50 nm for the surface area, pore volume and pore diameter. Importantly, the BET surface area of the MWCNTs-KIAgNPs was less than the surface area of 1250 m^2^/g reported for MWCNTs in our previous studies^33^. The observed reduction in the surface properties of the nano adsorbent could be due to the dense occupation of the tubular linkages of the MWCNTs by decoration with KIAgNPs. In the light of the observed decrease in surface area of MWCNTs-KIAgNPs after decoration with KIAgNPs, it was also reported that surface area of GnZVI/PAC and MWCNTs@SiO_2_-NH_2_ reduced after the incorporation of GnZVI and SiO_2_-NH_2_^56,57^.

The FTIR spectrum of the MWCNTs-KIAgNPs before and after adsorption of Cd(II), Cr(VI), Fe(II) and Ni(II) ions is shown in Fig. 2b. As can be seen in Fig. 2b, characteristic peaks were assigned to the following absorption bands: 895 (C–H), 1250 (C=O), 1959 (C≡C), 2114 (C≡C), 2365 (C≡C) and 2835 (C–H) cm^−1^, respectively^38^. The observed peaks were attributed to the bending and stretching due to alkene, alkane, alkyne and phenolic groups. Also, the observed absorption bands on MWCNTs-KIAgNPs before and after adsorption at 1078 and 3000 cm^−1^ were attributable to the vibrational stretching of C–O and O–H due to alkanes and carboxylic groups^58^. Noticeably, a very strong peak peculiar to KIAgNPs was found at 1700 cm^−1^ which corresponds to C=O stretching vibration of the amine^37^. Also, the peak at 1400 cm^−1^ could be attributed to C=C stretching and N–H vibrations phenolic and amine groups contained in the KI extract^38^. Notably, the peak at 3680 cm^−1^ on MWCNTs-KIAgNPs before and after adsorption were assigned to the O–H stretching frequencies of the hydroxyl group^38^. Remarkably, Fig. 2b revealed two distinctive peaks on MWCNTs-KIAgNPs prior to adsorption at 3890 cm^−1^ which corresponds to the stretching modes overlap of correlative amines, free amines and hydroxyl groups (NH_2_, –NH– and –OH)^42^. Also, the peak at 704 cm^−1^ could be attributed to C-H bend due to alkene. However, the disappearance of absorption bands at 704 and 3890 cm^−1^ on the MWCNTs-KIAgNPs after adsorption could be attributed to the attached metal ions on the surface of the nano adsorbent.

In this study, FTIR analysis validated that Ag^+^ reduction occurred due to various functional groups: flavonoids, tannins, phenols, terpenoids and polysaccharides present in the KI extract^37^. Furthermore, carbonyl groups bind to metals better than amino acids and proteins which principally forms coating layer on the metal nanoparticles surface, thereby suitably inhibiting accumulation and improving KIAgNPs stability^58^. More still, amine (–NH), carboxyl (–C=O) and hydroxyl (-OH) groups constituents of KI extract enhances the stability of KIAgNPs^32^.

To further examine the elemental and surface oxidation states of the nano adsorbent composite, XPS analysis was conducted and the result shown in Fig. 2c,d. Notably, Fig. 2c of MWCNTs-KIAgNPs clearly illustrated the appearance of three pronounced peaks at 284, 374 and 530 eV, indicating the nano adsorbent was predominantly composed of elemental C, Ag and O^27^. Likewise, the deconvolution of C (Is) spectrum presented in Fig. 2d revealed four peaks at 284, 286, 287.5 and 288.7 eV corresponding to C–C, C–OH, C=O and O–C=O bonds^26^. The XPS analysis showed good agreement with the FTIR (Fig. 2b) result, thereby confirming the presence of the reported functional groups and validating the observed presence of the incorporated materials onto the fabricated MWCNTs-KIAgNPs.

### Batch adsorption performance

#### Effect of solution pH

**Figure 3:**
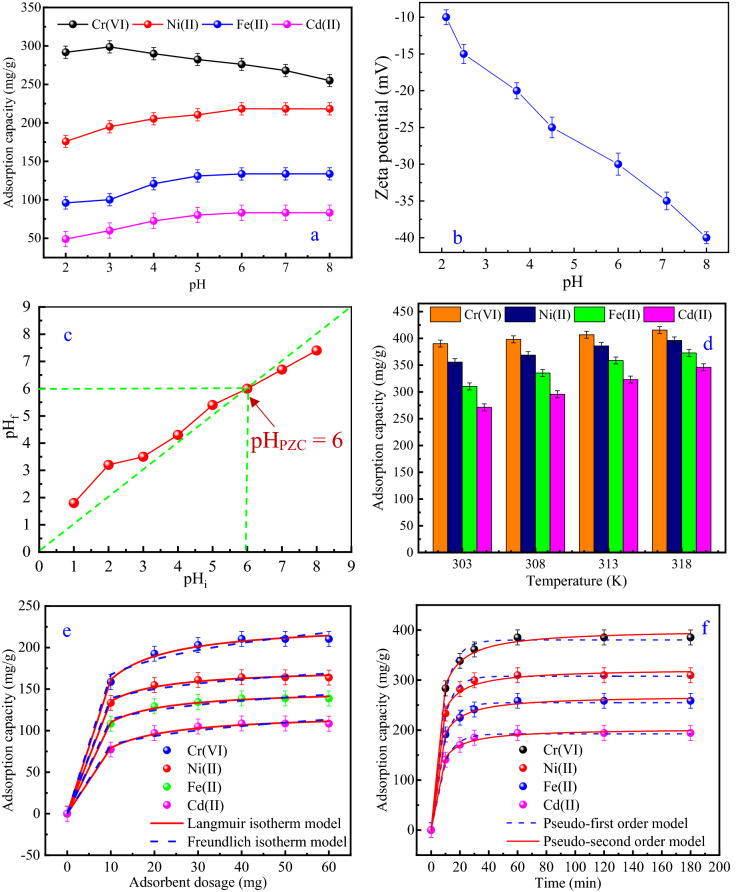
**(a)** Effect of pH on the adsorption of metal ions by MWCNTs-KIAgNPs; **(b)** point of zero charge pH (pH_PZC_) of MWCNTs-KIAgNPs; **(c)** zeta potentials of MWCNTs-KIAgNPs; **(d)** influence of temperature on the uptake of Cr(VI), Ni(II), Fe(II) and Cd(II) ions by MWCNTs-KIAgNPs; **(e)** Langmuir and Freundlich isotherm model curves of MWCNTs-KIAgNPs adsorption towards Cr(VI), Ni(II), Fe(II) and Cd(II); **(f)** pseudo-first order and pseudo-second order kinetic model for Cr(VI), Ni(II), Fe(II) and Cd(II) adsorption by MWCNTs-KIAgNPs.

Due to the interactions of the metal ions and adsorbents surface property in aqueous conditions^59^, it is important to examine the influence of solution pH on the adsorption of Cr(VI), Ni(II), Fe(II) and Cd(II) on MWCNTs-KIAgNPs. As presented in Fig. 3a, it is clearly evident that the adsorption effectiveness of MWCNTs-KIAgNPs towards Ni(II), Fe(II) and Cd(II) ions increased with increasing pH from 2 to 6, while Cr(VI) increased from 2 to 3. The adsorbed amount of Cr(VI) on the adsorbent further reduced with increase in pH above 3.

As can be seen from the figure, further increment in the removal of Cd(II), Ni(II) and Fe(II) were not noticeable beyond pH 6. Overall, the maximum adsorption capacity of Cd(II), Fe(II) and Ni(II) were obtained as 83.20, 133.63 and 218.32 mg/g, respectively at the pH of 6. On the other hand, the maximum adsorption capacity of Cr(VI) was obtained as 298.12 mg/g at the pH of 3. As reported previously, ${\mathrm{HCrO}}_{4}^{-}$, ${\mathrm{CrO}}_{4}^{2-}$, ${{\mathrm{Cr}}_{2}\mathrm{O}}_{7}^{2-}$, ${{\mathrm{Cr}}_{3}\mathrm{O}}_{10}^{2-}$ and ${{\mathrm{Cr}}_{4}\mathrm{O}}_{13}^{2-}$ are the various forms of chromium^60^ in a solution. Meanwhile, ${\mathrm{HCrO}}_{4}^{-}$ and ${{\mathrm{Cr}}_{2}\mathrm{O}}_{7}^{2-}$ are the dominant form of Cr(VI) in the solution at low pH, while ${\mathrm{CrO}}_{4}^{2-}$ are present at higher pH^61^. The higher removal of Cr(VI) at low pH (3) may be attributed to increase in ionizing ability of Cr(VI), formation of ${\mathrm{HCrO}}_{4}^{-}$ and positive surface formation^62^. Therefore, enhanced electrostatic attraction between the positively charged surface of MWCNTs-KIAgNPs in acidic medium and ${\mathrm{HCrO}}_{4}^{-}$ species was generated, which favored higher Cr(VI) adsorption. However, at higher pH the ${\mathrm{HCrO}}_{4}^{-}$ transformed to ${\mathrm{CrO}}_{4}^{2-}$ which resulted to reduce amount of Cr(VI) due to force of repulsion between the ${\mathrm{CrO}}_{4}^{2-}$ and negatively charged adsorbent surface. The decrease in Cd(II), Fe(II) and Ni(II) adsorption at low pH values may be due to the fierce adsorption competition between the highly positive H^+^ and the metal ions (Cd(II), Fe(II) and Ni(II)) for the occupation of the functional adsorption sites. Most importantly, the reduction in negative surface charge of MWCNTs-KIAgNPs may be due to protonation which enhanced the adsorbents positively charged surface, thereby correspond to decrease in electrostatic attraction towards Ni(II), Fe(II) and Cd(II) adsorption. However, the deprotonation phenomenon of MWCNTs-KIAgNPs surface at high pH resulted in increased negative charge of the adsorbent which enhanced the electrostatic interaction leading to maximum adsorption capacity. Correspondingly, the electrophoretic performance and surface charge of the MWCNTs-KIAgNPs were obtained by the zeta potential as shown in Fig. 3b. Importantly too, Fig. 3c revealed that the pH point of zero charge (pH_PZC_) of MWCNTs-KIAgNPs was obtained as 6. This indicates that the surface of MWCNTs-KIAgNPs was positively charged at pH_PZC_ < 6, while the adsorbent surface was negatively charged at pH_PZC_ > 6^15^. In all, pH 6 was selected for further investigation of Cd(II), Ni(II) and Fe(II) adsorption, while pH 3 was utilized for Cr(VI) adsorption due to its optimal adsorption values for all the metal ions without precipitation to metal hydroxides^59^. Similar pH impact on the adsorption of Cr(VI), Ni(II), Fe(II) and Cd(II) ions have been previously reported over the literature^61,63,64^.

#### Effect of temperature

The influence of temperature on Cr(VI), Ni(II), Fe(II) and Cd(II) ions adsorption by MWCNTs-KIAgNPs was examined at 303 to 318 K. As shown in Fig. 3d, the adsorption capacities were observed to increase with increasing temperatures and optimum adsorption capacities of Cr(VI) (416 mg/g), Ni(II) (396 mg/g, Fe(II) (372 mg/g) and Cd(II) (346 mg/g) were achieved at the temperature of 318 K. The favorability of the metal ions capture by MWCNTs-KIAgNPs may be linked to the endothermic reaction characteristics which reduced solution viscosity, increased external mass transfer and metal ions diffusion to the pores of the MWCNTs-KIAgNPs. Overall, metal ions adsorption were thermally governed due to activation energy generation by temperature thereby leading to aggregation ascribed to increase in kinetic energy and metal ions collision with the binding sites of the nano adsorbent^49^.

### Adsorption isotherm

The influence of MWCNTs-KIAgNPs dosage towards the effective adsorption of metal ions was investigated in the range of 10 to 60 mg/L in order to examine the contact areas between the adsorbent and metal ions. According to Fig. 3e, increase in metal ions adsorption capacity was noticed with increasing adsorbent dosage of 10 to 40 mg/L. Notably, the observed proportional increase in adsorption capacity with adsorbent dosage increase could be attributed to increased surface area of the MWCNTs-KIAgNPs and the abundant availability of adsorption sites. However, further increase in adsorbent dosage beyond 40 mg/L showed insignificant change in adsorption capacity which may be due to the saturation and over saturation of the adsorption sites^65^. Therefore, 40 mg/L was identified as the optimum amount of adsorbent for metal ions removal in this study. Importantly, higher adsorption capacity of 210.51, 164.26, 138.78 and 108.65 mg/g, respectively were obtained for Cr(VI), Ni(II), Fe(II) and Cd(II) adsorption by MWCNTs-KIAgNPs.

Subsequently, Langmuir and Freundlich isotherm model were used to fit the correlative data to assess the characteristic interface and MWCNTs-KIAgNPs affinity towards Cr(VI), Ni(II), Fe(II) and Cd(II) adsorption as revealed in Fig. 3e. Accordingly, a rapid-slow trend in adsorption was evident signaling that the availability of large adsorption sites could enhance adsorption capacity, while slower adsorption was attributed to the presence of limited adsorption sites^66^.

The isotherm parameters from the Langmuir and Freundlich models were calculated and the various values presented in Table 3. The removal of the metal ions was better fitted to Langmuir model due to the higher correlation coefficient closer to 1 (R^2^ = 0.971, 0.962, 0.967 and 0.965 for Cr(VI), Ni(II), Fe(II) and Cd(II) adsorption) and lower *X*^2^ values (11.946, 5.563, 4.621 and 5.330 for Cr(VI), Ni(II), Fe(II) and Cd(II)). Accordingly, a homogenous monolayer coverage of the Cr(VI), Ni(II), Fe(II) and Cd(II) ions on the surface of MWCNTs-KIAgNPs were dominant in the chemisorption controlled adsorption process, while the higher correlation coefficient validated the electrostatic interaction mechanism^42^.

**Table 3:** Calculated isotherm model parameters for metal ions adsorption by MWCNTs-KIAgNPs.

Adsorbate	q_e, exp_ (mg/g)	Langmuir isotherm model parameters					Freundlich isotherm model parameters			
		q_m_(mg/g)	K_L_ (L min^−1^)	R_L_	R^2^	X^2^	K_F_ (mg/g)	n_F_	R^2^	X^2^
Cr(VI)	210.513 ± 0.001	229.540 ± 0.001	0.237 ± 0.002	0.047 ± 0.003	0.971	11.946	119.203 ± 0.002	6.777 ± 0.001	0.844	64.355
Ni(II)	164.256 ± 0.002	174.784 ± 0.006	0.342 ± 0.003	0.042 ± 0.001	0.962	5.563	108.404 ± 0.001	9.240 ± 0.002	0.814	27.169
Fe(II)	138.775 ± 0.003	149.552 ± 0.005	0.279 ± 0.001	0.060 ± 0.002	0.967	4.621	84.465 ± 0.003	7.747 ± 0.001	0.833	23.662
Cd(II)	108.650 ± 0.002	121.026 ± 0.003	0.189 ± 0.004	0.108 ± 0.001	0.965	5.330	55.186 ± 0.001	5.691 ± 0.002	0.838	24.838

Of particular attention is that the adsorption intensity (n_F_) of all the metal ions were greater than 1, indicating possible favorability of metal ions uptake. Furthermore, the calculated maximum adsorption capacities were obtained as 229.540, 174.784, 149.552 and 121.026 mg/g for Cr(VI), Ni(II), Fe(II) and Cd(II) ions, respectively. Additionally, the preferential adsorption and the enhanced adsorption capacity of some of the metal ions over others could be linked to the differences in their ionic radius. For instance, the ionic radius of the metal ions are Cr(VI) (0.44 Å), Ni(II) (0.56 Å), Fe(II) (0.64 Å) and Cd(II) (0.78 Å)^67–69^. It is apparent that metals with smaller ionic radius diffuse faster onto the binding sites of the adsorbent, compared with metals with higher ionic radius^67^. Based on the ionic radius, the superior adsorption of the metal ions onto MWCNTs-KIAgNPs were in the range Cr(VI) > Ni(II) > Fe(II) > Cd(II).

### Adsorption kinetics

Further investigation of time-dependent curves of Cd(II), Cr(VI), Ni(II) and Fe(II) ions adsorption by MWCNTs-KIAgNPs were carried out in the range of 10–180 min as shown in Fig. 3f. Obviously, a rapid increase in the adsorption capacities of the metal ions were observed in the first 60 min which was ascribed to the increased diffusion of the metal ions to the rich adsorption sites and the presence of strong attractive forces between the metal ions and MWCNTs-KIAgNPs^70^. Subsequently, the adsorption capacity decreased gradually with further increase in time beyond 60 min due to the depletion of the binding sites^65^.

Herein, the experimental data were fitted by pseudo-first order and pseudo-second order kinetics as shown in Fig. 3f, while the parameters obtained from the kinetic equations are presented in Table 4. Based on the higher correlation coefficient (R^2^) and lower error (*X*^2^) values, pseudo-second order kinetic equation suitably fit the adsorption data for Cr(VI), Ni(II), Fe(II) and Cd(II) ions. Notably, the adsorption of Cr(VI), Ni(II), Fe(II) and Cd(II) ions corresponding to pseudo-second order kinetics clearly affirms that the adsorption of the metal ions by MWCNTs-KIAgNPs were dominated majorly by chemisorption behavior^66^.

**Table 4:** Calculated kinetic model parameters for metal ions adsorption by MWCNTs-KIAgNPs.

Adsorbate	C_o_ (mg L^−1^)	q_e, exp_ (mg g^−1^)	Pseudo-first order kinetic model parameters				Pseudo-second order kinetic model parameters			
			k_1_ (min^−1^)	q_e, cal_ (mg g^−1^)	R^2^	X^2^	k_2_ (g·mg^−1^·min^−1^) × 10^–4^	q_e, cal_ (mg g^−1^)	R^2^	X^2^
Cr(VI)	85.00	385.220 ± 0.004	0.128 ± 0.002	401.24 ± 0.003	0.935	106.411	1.723 ± 0.001	380.384 ± 0.005	0.968	53.030
Ni(II)	66.00	309.696 ± 0.006	0.137 ± 0.005	322.689 ± 0.002	0.926	67.091	1.557 ± 0.003	307.780 ± 0.004	0.980	18.501
Fe(II)	56.00	258.580 ± 0.003	0.128 ± 0.001	268.69 ± 0.004	0.910	65.874	0.976 ± 0.004	264.989 ± 0.003	0.973	19.594
Cd(II)	44.00	194.150 ± 0.012	0.124 ± 0.003	203.419 ± 0.005	0.949	22.773	0.767 ± 0.002	192.137 ± 0.002	0.963	16.836

### Adsorption thermodynamics

To further evaluate the thermodynamic parameters of Cr(VI), Ni(II), Fe(II) and Cd(II) ions adsorption by MWCNTs-KIAgNPs, the thermodynamic plots and evaluated parameters are shown in Fig. S2 and Table 5, respectively.

**Table 5:** Thermodynamic parameters for Cr(VI), Ni(II), Fe(II) and Cd(II) adsorption by MWCNTs-KIAgNPs.

Adsorbate	Temperature (K)	∆Gº (kJ/mol)	∆Hº (kJ/mol)	∆Sº (J/mol)
Cr(VI)	303	− 1155.28 ± 0.1	2153.33 ± 10.00	124.34 ± 4.00
	308	− 1777.00 ± 0.003		
	313	− 2920.44 ± 0.06		
	318	− 4163.88 ± 0.02		
Ni(II)	303	− 934.14 ± 0.04	1956.03 ± 9.20	118.61 ± 3.00
	308	− 1592.18 ± 0.02		
	313	− 2808.25 ± 0.06		
	318	− 4024.32 ± 0.05		
Fe(II)	303	− 875.14 ± 0.03	1787.34 ± 7.50	111.50 ± 2.00
	308	− 1367.64 ± 0.01		
	313	− 2552.63 ± 0.07		
	318	− 3637.63 ± 0.04		
Cd(II)	303	− 852.14 ± 0.02	1706.70 ± 6.10	102.35 ± 1.00
	308	− 1363.91 ± 0.04		
	313	− 2387.45 ± 0.03		
	318	− 3410.98 ± 0.01		

The results presented in Table 5 revealed negative values of ΔG° at different temperatures, indicating spontaneity of metal ions removal by MWCNTs-KIAgNPs. Correspondingly, the observed decrease of ΔG° values with temperature increase suggests enhanced adsorption at higher temperatures. Additionally, the metal ions adsorption was endothermic in nature due to the positive values of ΔH° indicating spontaneity increase and interaction with MWCNTs-KIAgNPs. Notably, the obtained values of ΔH° Cr(VI) (2153.33), Ni(II) (1956.03), Fe(II) (1787.34) and Cd(II) (1706.70) were higher than 40 kJ/mol, thereby confirmed that the adsorption process was chemisorption controlled^71^. Moreso, the ΔS° positive values Cr(VI) (124.34), Ni(II) (118.61), Fe(II) (111.50) and Cd(II) (102.35) revealed increasing randomness at Cr(VI)/Ni(II)/Fe(II)/Cd(II)-MWCNTs-KIAgNPs solution interface.

### Comparison with other adsorbents

Additionally, the adsorption capacities of the metal ions obtained in this study have been compared with previous studies as shown in Table 6. Remarkably, Table 6 revealed that MWCNTs-KIAgNPs showed higher adsorption capacity compared to the adsorption capacities by the various adsorbents from the literature. The observed higher adsorption capacity in this study could be attributed to the high surface area and functional groups of MWCNTs-KIAgNPs that provided massive binding sites for the adsorption of the metal ions. Although a higher surface area of 1500 m^2^/g were reported for PSBAC, but the adsorption capacity of 41.66 mg/g for Fe(II) removal was relatively small compared to the result of this study^72^. The observed differences may be due to the variations in the functional groups and structural nature of the materials.

**Table 6:** Comparison of adsorption parameters among the adsorbates and various adsorbents.

Adsorbent	Adsorbate	pH	Temp. (K)	Time (min)	Surface area (m^2^/g)	q_m_ (mg/g)	Isotherm/kinetics	References
α-Fe2O3@C	Cr(VI)	3	298	360	ND	76.92	Langmuir/PSO	Trang et al.^60^
GnZVI/PAC	Cr(VI)	2	323	80	107	53.48	Langmuir/PSO	Khosravi et al.^57^
DWHR	Cr(VI)	3	298	120	ND	1.28	Freundlich/PSO	Kumar and Chauhan^73^
CES	Ni(II)	6.5	298	960	ND	163.6	Langmuir/PSO	Peng et al.^74^
Goethite	Ni(II)	9	363	60	5.12	0.943	Langmuir/PSO	Dash et al.^63^
BTMC	Ni(II)	5	298	30	209	57.14	Langmuir/ND	Anitha et al.^75^
Ca-Pal	Fe(II)	6	298	10	297	3.71	Langmuir/ND	Lazaratou et al.^64^
Sepiolite	Fe(II)	5	293	30	200	12	Langmuir/ND	Kocaoba^76^
PSBAC	Fe(II)	3	303	90	1500	41.66	Temkin/PSO	Kaveeshwar et al.^72^
SLMO	Cd(II)	6	298	720	17.09	26.24	Langmuir/PSO	Zhang et al.^62^
Fe-BTC	Cd(II)	7	318	20	289.33 ± 1.60	9.55	Langmuir–Freundlich/ND	Zhang et al.^70^
CABs-MO	Cd(II)	6.5	298	720	2.04 ± 0.002	63.6	Langmuir/PSO	Shim et al.^77^
MWCNTs-KIAgNPs	Cr(VI)	3	313	60	1236 ± 5.40	229.540 ± 0.001	Langmuir/PSO	This study
MWCNTs-KIAgNPs	Ni(II)	6	313	60	1236 ± 5.40	174.784 ± 0.006	Langmuir/PSO	This study
MWCNTs-KIAgNPs	Fe(II)	6	313	60	1236 ± 5.40	149.552 ± 0.005	Langmuir/PSO	This study
MWCNTs-KIAgNPs	Cd(II)	6	313	60	1236 ± 5.40	121.026 ± 0.003	Langmuir/PSO	This study

*PFO* pseudo-first order, *PSO* pseudo-second order, *ND* not determined.

### Column adsorption performance

Fixed bed adsorption in a continuous flow seemingly demonstrates strong favorability for enhanced practical application. In all, fixed bed model parameters determination may aid the industrial scale up of the adsorption process without further experimentation^47^.

Therefore, investigation of the capture capacity of MWCNTs-KIAgNPs for Cr(VI), Ni(II), Fe(II) and Cd(II) ions in a continuous flow adsorption was performed. More than that, parameters influencing the dynamic adsorption behavior were examined by varying the initial metal concentration, bed height and flow rate on the breakthrough curve. Thereafter, the simulation of the experimental data using Thomas and Adams-Bohart model were accomplished.

#### Effect of bed height, inlet concentration and flow rate

The influence of bed height (2, 4 and 6 cm) towards Cr(VI), Ni(II), Fe(II) and Cd(II) ions removal in dynamic adsorption system was examined as shown in Fig. S3(a, d, g and j), while the determined parameters are presented in Table 7. As observed from the figures, decrease in bed height accelerated the duration for the breakthrough and saturation, culminating to the early bed saturation. On the other hand, increase in bed height enhanced both the time for breakthrough and exhaustion due to the longer length and contact time of the mass transfer zone (MTZ) at each column ends^78^. Particularly, increasing the bed length resulted to increase in the quantity of MWCNTs-KIAgNPs, thereby providing more adsorption sites for Cr(VI), Ni(II), Fe(II) and Cd(II) ions uptake. Notably, improved metal ions adsorption was attained by increasing the bed length and may be attributed to the availability of enormous time for the metal ions to interact with the MWCNTs-KIAgNPs.

**Table 7:** Determined parameters of breakthrough curves and continuous adsorption models for Cr(VI), Ni(II), Fe(II) and Cd(II) adsorption by MWCNTs-KIAgNPs.

Adsorbate	C (mg/L)	Q (mL/min)	Z (cm)	t_b_ (min)	q_total_ (mg/g)	q_e_ (mg)	L_MTZ_ (cm)	Thomas model			Adams-Bohart model		
								K_TH_	q_o_	R^2^	K_AB_	N_o_	R^2^
**Cr(VI)**	21.25	5.00	2	480	84.163	33.665	1.762	0.00030	35.923	0.966	0.00016	302.724	0.700
	21.25	5.00	4	1200	125.438	44.799	3.872	0.00029	46.368	0.991	0.00012	291.053	0.768
	21.25	5.00	6	1600	158.625	52.875	5.040	0.00027	53.668	0.999	0.00011	134.208	0.832
	42.50	5.00	4	720	108.525	38.759	4.352	0.00031	40.901	0.978	0.00017	259.935	0.743
	85.00	5.00	4	480	84.750	30.268	3.995	0.00035	31.346	0.973	0.00024	223.623	0.751
	21.25	7.50	4	720	98.694	35.248	3.619	0.00032	38.197	0.990	0.00015	210.398	0.789
	21.25	10.00	4	480	75.975	27.134	3.010	0.00034	28.629	0.980	0.00019	187.707	0.776
**Ni(II)**	16.50	5.00	2	720	78.225	31.290	1.810	0.00029	35.106	0.964	0.00011	199.466	0.680
	16.50	5.00	4	1200	112.397	40.142	3.524	0.00027	42.543	0.993	0.00010	236.755	0.772
	16.50	5.00	6	1600	143.192	47.731	5.040	0.00026	48.119	0.997	0.00009	275.309	0.793
	33.00	5.00	4	720	101.705	36.323	4.348	0.00034	37.096	0.997	0.00013	229.454	0.887
	66.00	5.00	4	480	72.626	25.938	3.706	0.00038	26.427	0.995	0.00015	208.611	0.748
	16.50	7.50	4	720	89.094	31.819	3.219	0.00030	34.341	0.992	0.00016	215.199	0.795
	16.50	10.00	4	240	68.630	24.511	2.914	0.00032	27.891	0.989	0.00019	172.134	0.773
**Fe(II)**	14.00	5.00	2	720	70.320	28.128	1.810	0.00022	26.179	0.981	0.00008	176.642	0.764
	14.00	5.00	4	1400	103.050	36.804	3.524	0.00021	35.597	0.992	0.00007	192.067	0.764
	14.00	5.00	6	1800	125.740	41.913	5.280	0.00020	41.792	0.999	0.00006	219.307	0.817
	28.00	5.00	4	960	94.980	33.921	3.876	0.00023	44.171	0.985	0.00009	191.292	0.723
	56.00	5.00	4	480	67.804	24.216	3.349	0.00026	25.900	0.976	0.00011	164.414	0.681
	14.00	7.50	4	720	86.948	31.053	3.519	0.00022	32.103	0.973	0.00008	176.618	0.719
	14.00	10.00	4	240	70.020	22.007	3.114	0.00024	23.417	0.973	0.00012	153.409	0.732
**Cd(II)**	11.00	5.00	2	720	64.320	25.728	1.810	0.00020	26.702	0.975	0.00005	105.026	0.777
	11.00	5.00	4	1400	92.625	33.080	3.658	0.00019	35.951	0.986	0.00006	167.225	0.801
	11.00	5.00	6	1800	114.055	38.018	5.280	0.00017	40.262	0.992	0.00007	200.772	0.831
	22.00	5.00	4	960	77.330	27.618	3.962	0.00022	27.919	0.988	0.00008	185.979	0.770
	44.00	5.00	4	480	59.260	21.164	3.231	0.00026	22.431	0.990	0.00010	155.660	0.749
	11.00	7.50	4	960	68.493	24.462	3.380	0.00021	25.451	0.983	0.00009	163.725	0.786
	11.00	10.00	4	720	54.520	19.471	2.810	0.00024	17.574	0.982	0.00012	148.019	0.663

The calculated parameters in Table 7 indicate that the maximum adsorption capacities (q_e_) for Cr(VI), Ni(II), Fe(II) and Cd(II) ions adsorption using bed length of 6 cm were obtained as 52.875. 47.731, 41.913 and 38.018 mg/g, respectively. Importantly, the trend of bed length impact on dynamic adsorption in this study showed strong collaboration with the literature on the increment of total adsorbing surface area of MWCNTs-KIAgNPs using highest bed length due to abundant binding sites for adsorption^78,79^. Comparatively, the obtained continuous adsorption capacities in this study were relatively lower than the values in batch process and may be ascribed to the limitations of mass transfer provided by fixed bed against complete mixing of a batch reactor. Likewise, the preferential adsorption in the order of Cr(VI) > Ni(II) > Fe(II) > Cd(II) may be due to the atomic radii of the metal ions.

The influent concentrations of Cr(VI), Ni(II), Fe(II) and Cd(II) ions were varied as demonstrated in Table 7. Accordingly, Fig. S3(b, e, h and k) illustrates the steepness of the breakthrough curves gradient with higher influent concentration of the metal ions. Of specific emphasis is the clear reduction in breakthrough time (*t*_b_) with increasing influent concentration as shown in Table 7. As expected, the observed behavior may be credited to the overwhelming driving force provided by the higher inlet concentration difference in order for the adsorption process to overpower the resistance of mass transfer^47^. Apparently, shorter exhaustion time was also evident as the influent concentration of the metal ions increased leading to faster saturation of the binding sites^78^. In other words, increase in influent metal concentration obviously enhanced the removal rate of the metal ions due to the corresponding increase in the driving force for mass transfer^79^. Remarkably, Table 7 reveals that the optimal column adsorption capacity (q_e_) of Cr(VI) (44.799 mg/g), Ni(II) (40.142 mg/g), Fe(II) (36.804 mg/g) and Cd(II) (35.080 mg/g) were obtained at low influent concentrations of Cr(VI) (21.25 mg/L), Ni(II) (16.50 mg/L), Fe(II) (14.00 mg/L) and Cd(II) (11.00 mg/L). The findings of this study showed consistency with the previously reported literature on continuous flow adsorption system and notably revealed that fixed bed adsorption is inlet concentration dependent^78,79^.

Further investigation on the influence of flow rate (5, 7.5 and 10 mL/min) on the breakthrough curve was examined for Cr(VI), Ni(II), Fe(II) and Cd(II) ions adsorption by MWCNTs-KIAgNPs as shown in Fig. S3(c, f, i and l). All in all, metal ions had ample time for interaction with MWCNTs-KIAgNPs at lower flow rates culminating to the observed higher removal capacity of Cr(VI), Ni(II), Fe(II) and Cd(II) ions^47^. As demonstrated through the determined parameters in Table 7, increase in flow rate from 5 to 10 mL/min led to clear decrease in the adsorption capacity (q_e_) of metal ions from 44.799 to 27.134 mg/g for Cr(VI), 40.142 to 24.511 mg/g for Ni(II), 36.804 to 22.007 mg/g for Fe(II) and 33.080 to 19.471 mg/g for Cd(II). Not only that, the maximum total adsorbed amount (q_total_) of Cr(VI), Ni(II), Fe(II) and Cd(II) ions were 125.438, 112.397, 103.050 and 92.625 mg, respectively on the completion of the exhaustion time. It can be seen that the observed decrease in adsorption capacity was due to incomplete adsorption attributed to insufficient contact time of the aqueous phase in the fixed bed and the metal ions diffusion into the nanopores of MWCNTs-KIAgNPs^42^. Overall, lower flow rate favored Cr(VI), Ni(II), Fe(II) and Cd(II) ions adsorption owing to the higher uptake efficiency at lower flow rate. Previous studies have reported lower flow rate with a corresponding higher adsorption capacity^47,79^.

### Modelling of dynamic column

The prediction of the breakthrough curve for Cr(VI), Ni(II), Fe(II) and Cd(II) ions dynamic adsorption by MWCNTs-KIAgNPs surface was fitted using Thomas and Adams-Bohart model. Generally, Thomas model correlates with Langmuir isotherm and pseudo-second order kinetics assuming ideal model without axial diffusion, while Adams-Bohart model assumes non instantaneous balance where the rate of adsorption is dependent on remaining concentrations of metal ions and adsorption capacity of MWCNTs-KIAgNPs^47^. The calculated parameters of Thomas and Adams-Bohart model are presented in Table 7. Comparison of the fitness of dynamic adsorption data with the models were based on the higher values of R^2^, Consequently, Table 7 shows that the experimental data were best fitted by Thomas model with R^2^ above 0.964, compared to the Adams-Bohart model with the highest R^2^ of 0.887, thereby corresponding to pseudo-second order rate driving force^80^. Above all, the results obtained for the continuous adsorption corresponds to results of batch adsorption based on assumption of Thomas model for pseudo-second order kinetics which remarks that adsorption process was mass transfer controlled at the interface, not by chemical reaction^49^. In addition, Table 7 reveals that the determined data (q_o_) from Thomas model showed strong closeness to the experimental data (q_e_). In all dynamic adsorption, the K_TH_ which describe the transfer rate of metal ions was enhanced by higher metal ions inlet concentration, higher flow rate and lower bed depth, indicating the dominance of the overall kinetics by the external mass transfer. More so, K_TH_ provided the following adsorption order Cr(VI) > Ni(II) > Fe(II) > Cd(II) which may be explained by the variations in the metallic ionic radii and physicochemical properties of metal ions^81^. Similarly, it can be seen that higher metal ions inlet concentration, higher flow rate and lower bed depth resulted to lower values of q_o_ and may be linked to the insufficient contact time between the metal ions and MWCNTs-KIAgNPs. Therefore, the adsorption efficiency of MWCNTs-KIAgNPs towards Cr(VI), Ni(II), Fe(II) and Cd(II) ions in dynamic column is optimally improved by lower inlet concentration, lower flow rate and higher bed depth.

### Physico-chemical characteristics of the treated water

The investigation of the physico-chemical parameters metal ion analysis revealed the acidic nature of the chemical industry wastewater and high compositions of chromium, nickel, iron, cadmium, TDS, BOD, COD, nitrate, chloride, phosphate, fluoride and sulphate above the permissible standard concentrations recommended by US, Environmental Protection Agency and World Health Organization^82,83^. As can be seen in Table 8, the adsorption of the wastewater by MWCNTs-KIAgNPs significantly reduced the pollution parameters to fall within the threshold concentrations of the standards. The observed reduction could be attributed to the attachment of the pollutants to the functional groups and massive adsorption sites of the nano adsorbent^50^. In view of the results, the obtained treated water in both batch and column adsorption could be reused for various household and industrial purposes.

**Table 8:** Physico-chemical parameters of chemical industry wastewater and treated water in batch and fixed bed column.

Parameters	Raw wastewater concentration	Concentration after treatment using MWCNTs-KIAgNPs		Permissible standards	
		Batch adsorption	Fixed bed adsorption	US, EPA^82^	WHO^83^
Chromium (mg/L)	85.00 ± 0.06	0.020 ± 0.006	0.030 ± 0.004	0.1	0.05
Nickel (mg/L)	66.00 ± 0.03	0.040 ± 0.005	0.020 ± 0.002	0.1	0.07
Iron (mg/L)	56.00 ± 0.02	0.200 ± 0.002	0.180 ± 0.003	0.50	0.3
Cadmium (mg/L)	44.00 ± 0.01	0.002 ± 0.001	0.001 ± 0.001	0.005	0.003
pH	3.90 ± 0.05	6.95 ± 0.75	6.80 ± 0.60	6.5–8.5	5.5–8.5
TDS (mg/L)	36,49 ± 30.50	455.00 ± 5.80	432.00 ± 7.50	500	600
BOD (mg/L)	123.00 ± 0.40	8.33 ± 0.50	9.43 ± 0.52	10	10
COD (mg/L)	24,615 ± 25.30	12.34 ± 0.10	16.00 ± 0.20	40	40
Nitrate (mg/L)	489.00 ± 0.25	23.81 ± 0.15	30.13 ± 1.10	40	50
Chloride (mg/L)	1,980.00 ± 8.20	65.00 ± 1.22	76.08 ± 1.50	200	250
Phosphate (mg/L)	190.40 ± 0.65	0.20 ± 0.04	0.10 ± 0.001	1	0.5
Fluoride (mg/L)	103.00 ± 5.40	1.30 ± 0.03	1.15 ± 0.02	2	1.5
Sulphate (mg/L)	2790.00 ± 2.70	81.03 ± 2.40	48.62 ± 1.20	200	250

### Adsorption mechanism

**Figure 4:**
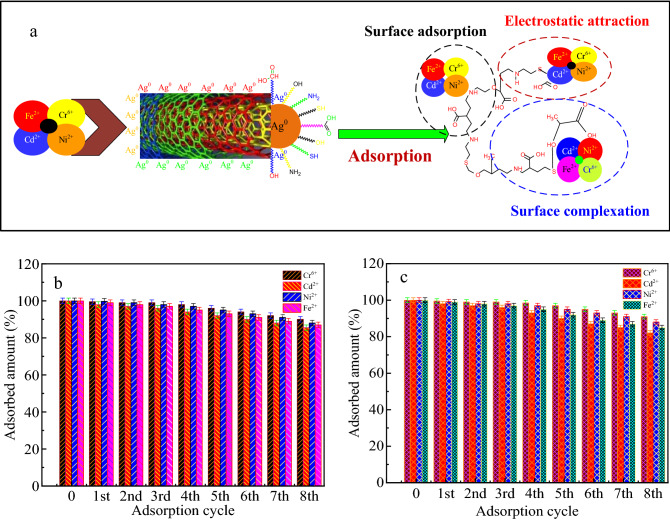
**(a)** possible adsorption mechanism of Cr(VI), Ni(II), Fe(II) and Cd(II) ions adsorption by MWCNTs-KIAgNPs; Reusability of MWCNTs-KIAgNPs towards Cr(VI), Ni(II), Fe(II) and Cd(II) ions adsorption in **(b)** batch system (MWCNTs-KIAgNPs dosage: 40 mg, pH 3 for Cr(VI) and 6 for Ni(II), Fe(II) and Cd(II) ions and contact time = 60 min) and **(c)** continuous system (bed depth = 6 cm, flow rate = 5 mL/min, influent concentration = Cr(VI) (21.25 mg/L), Ni(II) (16.50 mg/L), Fe(II) (14.00 mg/L) and Cd(II) (11.00 mg/L).

Through the analytical results of HRSEM (Fig. 1i) and FTIR (Fig. 2b) spectra, the possible mechanism of Cr(VI), Ni(II), Fe(II) and Cd(II) ions adsorption by MWCNTs-KIAgNPs shown in Fig. 4a was principally controlled simultaneously by supportive parameters including electrostatic interaction, surface adsorption and surface complexation. In view of the HRSEM information, the porous surface of MWCNTs-KIAgNPs enhanced the mass transfer and provided adsorption routes and area responsible for Cr(VI), Ni(II), Fe(II) and Cd(II) ions removal via diffusion to the pores and subsequent attachment to the surface of the adsorbent. However, the occurrence of electrostatic interaction was clearly evident between the positively charged metal (Cr(VI), Ni(II), Fe(II) and Cd(II)) ions and negatively charged MWCNTs-KIAgNPs adsorbent above the pH_PZC_.

In addition, the monolayer adsorption indicated dominance of the adsorption process in both batch and continuous process, validated by the Langmuir isotherm and Thomas model. Also, ion exchange and surface complexation were obvious in the combination of the positively charged metal ions and the active sites of negatively charged MWCNTs-KIAgNPs through electrostatic attraction, and subsequent removal via the carboxyl and hydroxyl groups^42^.

Incidentally, the FTIR spectra of MWCNTs-KIAgNPs before and after adsorption of metal ions failed to reveal obvious change, except for the disappearance of adsorption peaks matching 704 (C–H) and 3890 cm^−1^ (NH_2_, –NH– and –OH) after adsorption. This shows that the bonding of the metal ions with the hydroxyl groups were formed through surface complexation, whereas the hydroxyl/deprotonated groups could form complexes with Cr(VI), Ni(II), Fe(II) and Cd(II). Hence, the surface complexation mechanism occurred via metal ions deposition on the adsorbent-adsorbate interface and intermolecular interaction between the metal ions and the adsorbent^66^.

### Batch/column reusability

To further affirm the viability and longevity of the multi-functional MWCNTs-KIAgNPs for wastewater treatment, reusability was examined through adsorption–desorption techniques to validate the economical sustainability of the adsorbent. It is noteworthy that Cr(VI), Ni(II), Fe(II) and Cd(II) ions were remarkably recuperated from MWCNTs-KIAgNPs, affirming the applicability of the adsorbent for at least 8 cycles as shown in Fig. 4b,c.

It can be seen that the total adsorption capacity after 8 adsorption cycles in batch mode were Cr(VI) (91%), Ni(II) (89%), Fe(II) (87%) and Cd(II) (85.5%), respectively. Similarly, the adsorption capacity in the continuous adsorption system were revealed as Cr(VI) (90%), Ni(II) (88%), Fe(II) (85%) and Cd(II) (82%), respectively after the 8th adsorption cycle. It is noteworthy in column regeneration that reduction in saturation time was observed via progression of the regeneration process from first to eight adsorption cycle, thereby resulting to reduce adsorption capacity and expanded mass transfer zone. Meanwhile, the decline in adsorption behavior of both batch and column process may be attributable to the protonation of the adsorption sites and weakening of continuously used functional groups. Apparently, the observed slight reduction in adsorption capacity may also be ascribed to the accumulation of metal ions in the stacked tubular structures hindering metal ions further access to the binding sites^65^. Therefore, MWCNTs-KIAgNPs adsorbent possess excellent adsorption capacity, high selectivity and extended reusability capacity for efficient and economical recovery of metal ions from wastewater in both batch and column mode.

## Conclusion

In summary, the synthesis of AgNPs demonstrated using *Khaya ivorensis* (KI) leaves extract for the fabrication of MWCNTs-KIAgNPs was reported for the first time and employed for the adsorption of Cr(VI), Ni(II), Fe(II) and Cd(II) ions from chemical industry wastewater in a static (batch) and dynamic (continuous) processes. In the batch adsorption study, maximum adsorption of metal ions by MWCNTs-KIAgNPs were recorded at adsorbent dosage (40 mg), pH (3 for Cr(VI) and 6 for Ni(II), Fe(II) and Cd(II) ions) and contact time (60 min). Based on the fitness of the experimental data, Langmuir isotherm and pseudo-second order kinetic model demonstrated the best fit, indicating a monolayer and chemisorption controlled process. Evidently, thermodynamic parameters revealed that the adsorption process was endothermic and spontaneous. Furthermore, fixed bed adsorption columns validated the effectiveness of metal ions adsorption by MWCNTs-KIAgNPs at various operational conditions of low influent concentration, low flow rate and high bed depth. Notably, the experimental data was nicely predicted by Thomas model, which showed corroboration with the Langmuir isotherm and pseudo-second order kinetics in batch mode. Remarkably, the batch/column reusability study demonstrated that the metal ions removal percentage were still over 80%, even after the 8^th^ adsorption cycle. Overall, this study indicates the potentials of MWCNTs-KIAgNPs for wastewater treatment processes and may open novel approach towards developing robust nano sorbents for environmental remediation and related applications.

## Supplementary Information

Supplementary Figures.

Supplementary Tables.
