# Supplementary material for: Adsorption of Cr(VI), Ni(II), Fe(II) and Cd(II) ions by KIAgNPs decorated MWCNTs in a batch and fixed bed process
Source: Sci Rep. 2021 Jan 8;11:75. doi: 10.1038/s41598-020-79857-z (PMC7794394; doi:10.1038/s41598-020-79857-z)
Supplement: Supplementary file 1 — Supplementary Figures. [file 41598_2020_79857_MOESM1_ESM.docx]

**Adsorption of Cr(VI), Ni(II), Fe(II) and Cd(II) ions by KIAgNPs decorated MWCNTs in a batch and fixed bed process.**

**Titus Chinedu Egbosiuba^a, b, f *^, Ambali Saka Abdulkareem^a, f^, Abdulsalami Sanni Kovo^a, f^, Eyitayo Amos Afolabi^a^, Jimoh Oladejo Tijani^c, f^, Mercy Temitope Bankole^c, f^, Shufeng Bo^d^ and Wiets Daniel Roos^e^**

^a^ Department of Chemical Engineering, Federal University of Technology, PMB.65, Minna, Niger State, Nigeria

^c^ Department of Chemistry, Federal University of Technology, PMB.65, Minna, Niger State, Nigeria

^b^ Department of Chemical Engineering, Chukwuemeka Odumegwu Ojukwu University, PMB 02, Uli, Anambra State, Nigeria

^d^ Faculty of Light Industry and Chemical Engineering, Dalian Polytechnic Univeristy, Dalian 116034, P. R. China

^e^ Department of Physics, University of the Free State, P.O. Box 339, ZA-9300 Bloemfontein, South Africa

^f^ Nanotechnology Research Group, Africa Centre of Excellence for Mycotoxin and Food Safety, Federal University of Technology, P.M.B 65, Bosso, Minna, Niger State, Nigeria

**Corresponding Author: T.C. Egbosiuba**

E-mail: [egbosiubachinedu@gmail.com](mailto:egbosiubachinedu@gmail.com), ct.egbosiuba@coou.edu.ng

Address: Department of Chemical Engineering, Chukwuemeka Odumegwu Ojukwu University, PMB 02, Uli, Anambra State, Nigeria

Telephone: +2348034641162

**Supplementary List of Table**

**Table S1. ANOVA of green synthesis of KIAgNPs.**

**Table S2. EDS Elemental composition of MWCNTs-KIAgNPs before adsorption.**

**Table S3. EDS Elemental composition of MWCNTs-KIAgNPs after adsorption.**

**Table S1**

**ANOVA of green synthesis of KIAgNPs.**

| **Parameter** | **Sum of Squares** | **DF** | **Mean Squares** | **Coefficient  Estimate** | **F Value** | **P-value** | **Remark** |
| --- | --- | --- | --- | --- | --- | --- | --- |
| A | 242.00 | 1 | 242.00 | 5.50 | 4.36 | < 0.0041 | significant |
| B | 924.50 | 1 | 924.50 | 10.75 | 16.77 | < 0.0015 | “ |
| C | 684.50 | 1 | 684.50 | -9.25 | 12.42 | < 0.0024 | “ |
| Error | 220.50 | 4 | 55.13 | 3.00 | - |  |  |
| Total | 2071.50 | 7 | - | - | - |  |  |

**Table S2**

**EDS Elemental composition of MWCNTs-KIAgNPs before adsorption.**

| **Element** | **(Atomic %)** |
| --- | --- |
| C | 55.13 |
| O | 16.23 |
| Ag | 28.64 |
| Total | 100 |

**Table S3**

**EDS Elemental composition of MWCNTs-KIAgNPs after adsorption.**

| **Element** | **(Atomic %)** |
| --- | --- |
| C | 30.02 |
| O | 5.37 |
| Ag | 20.50 |
| Cr(VI) | 14.16 |
| Ni(II) | 12.45 |
| Fe(II) | 9.39 |
| Cd(II) | 8.11 |
| Total | 100 |
