# Supplementary material for: Adsorption of Cr(VI), Ni(II), Fe(II) and Cd(II) ions by KIAgNPs decorated MWCNTs in a batch and fixed bed process
Source: Sci Rep. 2021 Jan 8;11:75. doi: 10.1038/s41598-020-79857-z (PMC7794394; doi:10.1038/s41598-020-79857-z)
Supplement: Supplementary file 2 — Supplementary Tables. [file 41598_2020_79857_MOESM2_ESM.docx]

**Adsorption of Cr(VI), Ni(II), Fe(II) and Cd(II) ions by KIAgNPs decorated MWCNTs in a batch and fixed bed process.**

**Titus Chinedu Egbosiuba^a, b, f *^, Ambali Saka Abdulkareem^a, f^, Abdulsalami Sanni Kovo^a, f^, Eyitayo Amos Afolabi^a^, Jimoh Oladejo Tijani^c, f^, Mercy Temitope Bankole^c, f^, Shufeng Bo^d^ and Wiets Daniel Roos^e^**

^a^ Department of Chemical Engineering, Federal University of Technology, PMB.65, Minna, Niger State, Nigeria

^c^ Department of Chemistry, Federal University of Technology, PMB.65, Minna, Niger State, Nigeria

^b^ Department of Chemical Engineering, Chukwuemeka Odumegwu Ojukwu University, PMB 02, Uli, Anambra State, Nigeria

^d^ Faculty of Light Industry and Chemical Engineering, Dalian Polytechnic Univeristy, Dalian 116034, P. R. China

^e^ Department of Physics, University of the Free State, P.O. Box 339, ZA-9300 Bloemfontein, South Africa

^f^ Nanotechnology Research Group, Africa Centre of Excellence for Mycotoxin and Food Safety, Federal University of Technology, P.M.B 65, Bosso, Minna, Niger State, Nigeria

**Corresponding Author: T.C. Egbosiuba**

E-mail: [egbosiubachinedu@gmail.com](mailto:egbosiubachinedu@gmail.com), ct.egbosiuba@coou.edu.ng

Address: Department of Chemical Engineering, Chukwuemeka Odumegwu Ojukwu University, PMB 02, Uli, Anambra State, Nigeria

Telephone: +2348034641162

**Supplementary List of Figures**

**Fig. S1. EDS of MWCNTs-KIAgNPs.**

**Fig. S2. Thermodynamic plots of (a) Cr(VI); (b) Ni(II); (c) Fe(II) and (d) Cd(II) adsorption by MWCNTs-KIAgNPs.**

**Fig. S3. Experimental and theoretical breakthrough curves for the adsorption of Cr(VI) ion at different (a) bed height; (b) inlet concentration; (c) flow rates; the adsorption of Ni(II) ion at different (d) bed height; (e) inlet concentration; (f) flow rates; the adsorption of Fe(II) ion at different (g) bed height; (h) inlet concentration; (i) flow rates and the adsorption of Cd(II) ion at different (j) bed height; (k) inlet concentration and (l) flow rates by MWCNTs-KIAgNPs.**

**Fig. S1. EDS of MWCNTs-KIAgNPs**

a

**Fig. S2. Thermodynamic plots of (a) Cr(VI); (b) Ni(II); (c) Fe(II) and (d) Cd(II) adsorption by MWCNTs-KIAgNPs.**

**Fig. S3. Experimental and theoretical breakthrough curves for the adsorption of Cr(VI) ion at different (a) bed height; (b) inlet concentration; (c) flow rates; the adsorption of Ni(II) ion at different (d) bed height; (e) inlet concentration; (f) flow rates; the adsorption of Fe(II) ion at different (g) bed height; (h) inlet concentration; (i) flow rates and the adsorption of Cd(II) ion at different (j) bed height; (k) inlet concentration and (l) flow rates by MWCNTs-KIAgNPs.**
